# Enaminones in Heterocyclic Synthesis: A Novel Route to Tetrahydropyrimidines, Dihydropyridines, Triacylbenzenes and Naphthofurans under Microwave Irradiation

**DOI:** 10.3390/molecules15010058

**Published:** 2009-12-25

**Authors:** S.M. Al-Mousawi, M.A. El-Apasery, M.H. Elnagdi

**Affiliations:** Department of Chemistry, Faculty of Science; Kuwait University, P.O. Box 5969 Safat, 13060, Kuwait

**Keywords:** enaminones, Biginelli's condensation, naphthofuran, zeolites, microwave irradiation with simultaneous cooling

## Abstract

Condensation of phthalimidoacetone (**1**) with DMFDMA (*N,N*-Dimethylformamide dimethyl acetal) has afforded enaminone **2**. Refluxing **2** with equimolecular amounts of benzaldehyde and urea in acetic acid afforded a mixture of tetrahydropyrimidine **5** and the dihydropyridine **6**. Compound **2** undergoes self-condensation on heating in acetic acid or under microwave irradiation in presence of acidic zeolite to give 1,3,5-triacylbenzene **9**. Reacting enaminone **11a** with naphthoquinone **15** afforded the naphthofuran **18**. The possible formation of the aldehyde **19** was excluded based on an HMQC experiment, which revealed that the carbonyl carbon is not linked to any hydrogen.

## Introduction

Enaminones are valuable intermediates in synthetic organic chemistry [1,2,3,4]. In the last few years we and others have reported a variety of syntheses of heteroaromatics that have been developed using functionally substituted enamines as readily obtainable building blocks possessing multi electrophilic and nucleophilic moieties [5,6,7,8,9]. In conjunction to this work we report here an efficient microwave assisted synthesis of 2-(4-dimethylamino-2-oxo-but-3-enyl)isoindole-1,3-dione (**2**) and the results of our investigations devoted to exploring its synthetic potential. The work has enabled new synthetic approaches to the title compounds. We report here on the utility of **2** as precursor to substituted naphthofurans. In the course of this research we observed that the enaminone reactions were rather sluggish at room temperature, affording the final naphthofuran in moderate yield., but during the course of our explorations, we observed that the rate and the yields of these reactions could be greatly improved when performed under microwave irradiation in combination with simultaneous cooling at 30 °C. In this contribution, we would like to disclose the preliminary results of our investigations.

## Results and Discussion

**Scheme 1:**
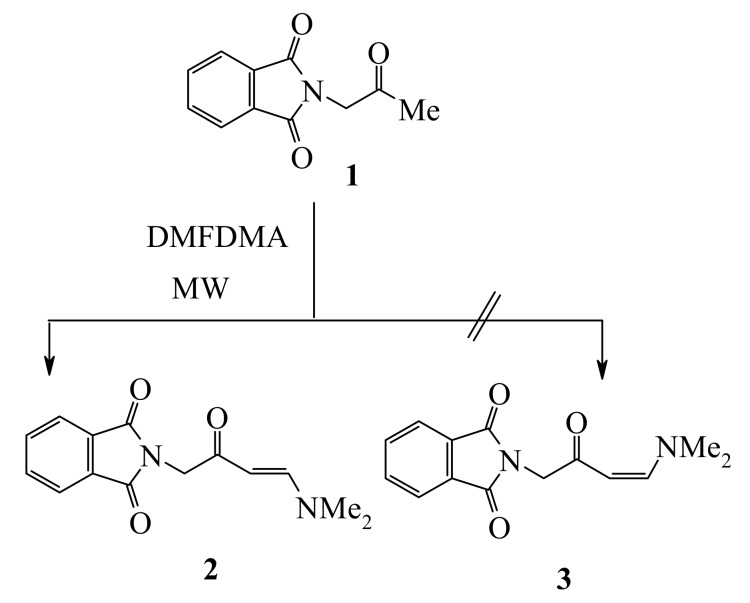
Preparation of 2-(4-dimethylamino-2-oxo-but-3-enyl)isoindole-1,3-dione (2).

Condensation of phthalimidoacetone (**1**) with dimethylformamide dimethyl acetal (DMFDMA) has afforded enaminone **2** in 76% and 77 % yields, respectively, either by refluxing in xylene for 8 h or by heating in MW without solvent at 180 °C for 20 min. The same condensation product had been obtained earlier by Al-Mousawi *et al.* [10,11]. Fischer *et al.* [12] have used coupling of carbonyl carbons with protons to establish relative geometry extensively, but using ^13^C-NMR to identify the long range coupling of C-H as an indication of the stereochemistry from the CO group is very difficult because of the appearance of carbonyl carbons as broad signals. This is expected theoretically because this carbonyl carbon has three different 2*J*, 2*J* and 3*J* couplings with nearby protons. We have run NOE difference experimentx to establish the stereo-orientation of either *E* or *Z*-forms for enaminone **2**. The NOE showed that irradiating the alkene CH at δ 5.04 ppm enhanced the dimethylamino protons at δ 2.79 and 3.08 ppm, confirming that they are proximal in space as required by the *E*-form (*cf.* Scheme 1).

**Scheme 2:**
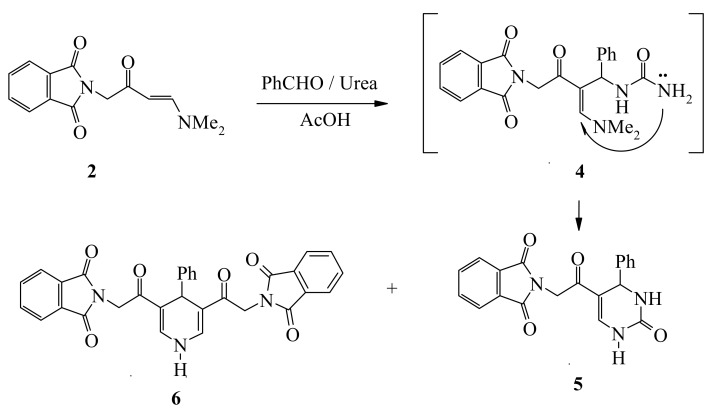
Preparation of tetrahydropyrimidine 5 and dihydropyridine 6.

Compound **2**, thus formed, has proven to be a versatile starting material for a variety of otherwise not readily obtainable functionally substituted aromatics and heteroaromatics. Thus, refluxing **2** with equimolecular amounts of benzaldehyde and urea in acetic acid afforded a 3:1 mixture of tetrahydropyrimidine **5** and the dihydropyridine **6**, which was readily separated by fractional crystallization (*cf.* Scheme 2).

**Scheme 3:**
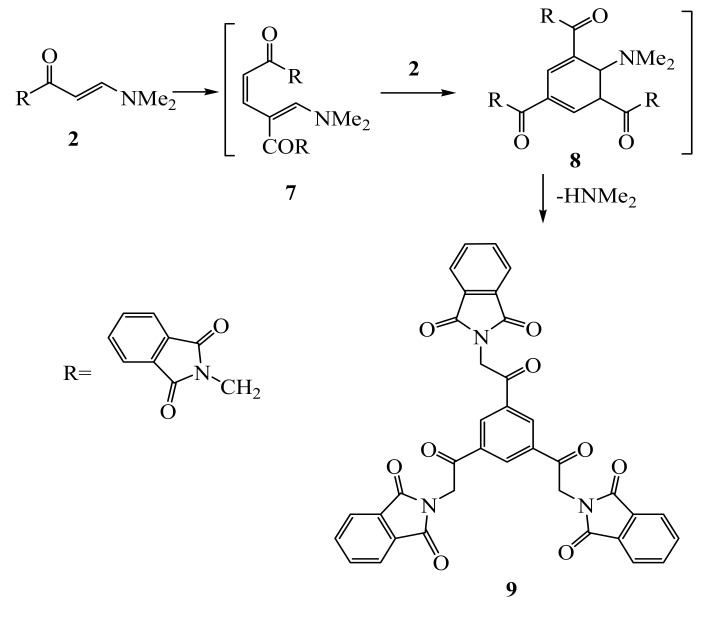
Synthesis of 2-(2-3,5-di[2-(1,3-dioxo-2,3-dihydro-1H-2-isoindolyl)acetyl]-phenyl-2-oxoethyl)-1,3-isoindolinedione (9).

Compound **2** undergoes self-condensation upon heating in acetic acid for 10 hours or upon irradiation at 150 ºC for 1 hour in a focused microwave oven in the presence of an acidic zeolite (Montmorillonite K-10) to give 1,3,5-triacylbenzene **9**. We believe that compound **2** is initially converted to the open chain intermediate **7** that further adds to one molecule of enaminone **2** to yield the intermediate **8**, which aromatize under these reaction conditions to give the final isolable product **9** (*cf.* Scheme 3).

**Scheme 4:**
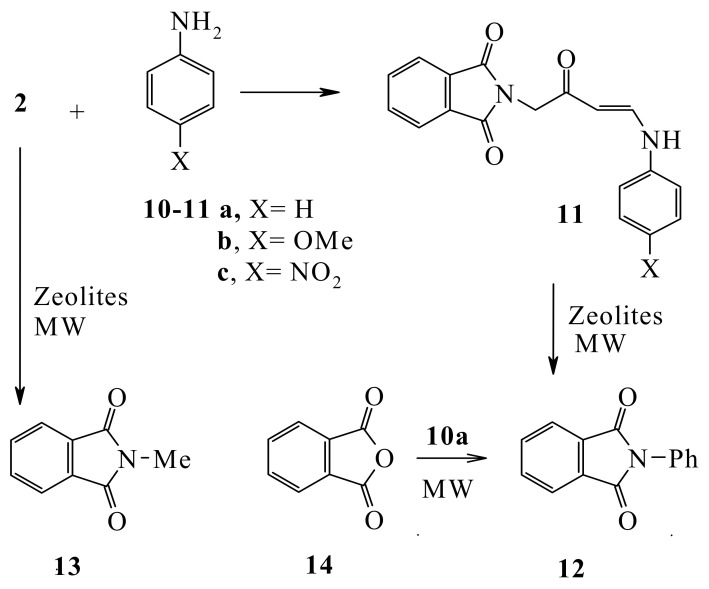
Synthesis of 2-(2-oxo-4-phenylamino-but-3-enyl)isoindole-1,3-dione, 2-[4-(4-methoxyphenylamino)-2-oxo-but-3-enyl]isoindole-1,3-dione, and 2-[4-(4-nitrophenyl-amino)- 2-oxo-but-3-enyl]isoindole-1,3-dione.

Compound **2** reacted with anilines **10a-c** to yield the aniline derivatives **11a-c**. Compound **11a** when heated with zeolite in an attempt to effect an intramolecular Friedel Craft’s cyclization reaction under MW in the absence of solvent at 160 °C for 30 min afforded *N*-phenylphthalimide (**12**) that was readily obtained from the MW assisted reaction of phthalic anhydride (**14**) with aniline without solvent at 180 °C for 60 min. It is most likely that in the initial step in this transformation the nitrogen atom lone pair attacks the phthalimidocarbonyl moiety. Similarly, heating (**2**) by MW in Zeolite without solvent at 160 °C for 30 min. afforded *N*-methylphthalimide (**13**) (*cf.* Scheme 4).

We decided to investigate the reaction utilizing microwave irradiation, while keeping the temperature at 30 °C. This should also allow maintaining the maximum power input of 80 W during the full run of the irradiation. To ensure a correct temperature measurement, a fiber optic sensor was used. Using the same reagent ratios and solvent as those for the reaction run at room temperature (~25 °C), we performed the reaction of enaminone **11a** with naphthoquinone **15** under microwave irradiation with simultaneous cooling at 30 °C.

The reaction proceeded smoothly, furnishing the corresponding naphthofuran **18**. The yield of the product was increased to 76%; The conventional method reaction at room temperature yielded only 68%. It is noteworthy that the reactions could be performed using an irradiation power of 80 W continuously during the whole run, as the high power level was needed to maintain the temperature at 30 °C because of the efficient external cooling. The differences in yield could be attributed to a lower rate of decomposition of the compounds when using simultaneous cooling.

The possibility of the formation of the aldehyde **19** was excluded based on the HMQC data which revealed that the carbonyl carbon is not linked to any hydrogen.

**Scheme 5:**
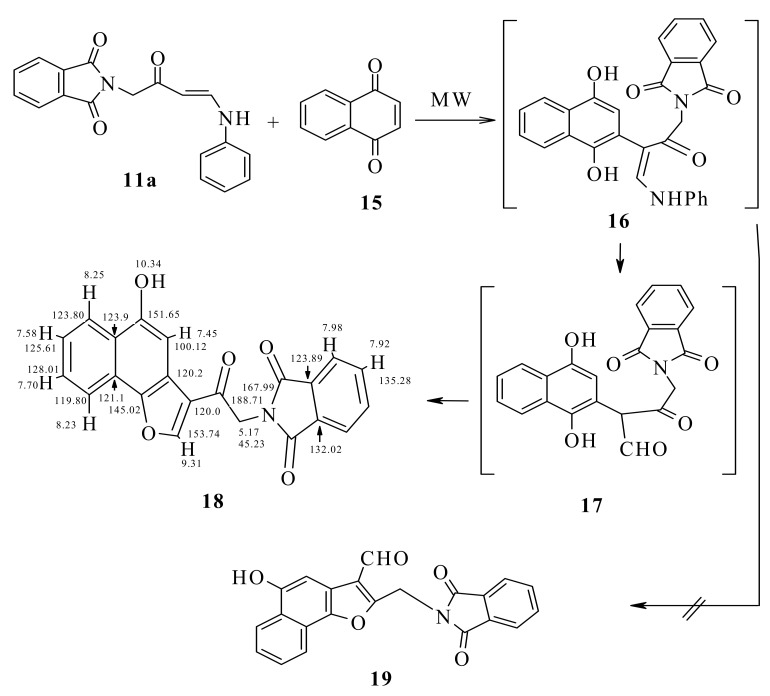
Synthesis of 2-[2-(5-hydroxynaphtho[1,2-b]furan-3-yl)-2-oxoethyl]isoindole-1,3-dione.

From H,H-COSY, H-8 at δ 7.70 correlates with H-9 at δ 8.23 and H-7 at δ 7.58 correlates with H-8 and with H-6 at δ 8.25 ppm. From HMBC, H-9 was detected at δ 8.23 ppm because it correlates with C-9b at δ 145.0, which also correlates with H-2 at δ 9.31 ppm. CO-12 correlated at δ 167.99 with CH_2_-11 at δ 5.70 ppm and CH-13 at δ 7.98 ppm (*cf.* Scheme 5).

## Experimental

### General

Melting points are uncorrected. All the reactions were conducted under microwave irradiation in heavy-walled Pyrex tubes (capacity 10 mL) fitted with PCS caps. Microwave heating was carried out with a single mode cavity Explorer Microwave Synthesizer (CEM Corporation, NC, USA), producing continuous irradiation and equipped with simultaneous external air-cooling system. Compound **17** was prepared in a microwave irradiation experiment which was carried out in a dedicated CEM-Discover-Coolmate monomode microwave apparatus operating at a frequency of 2.45 GHz with continuous irradiation power from 0 to 300 W. The reaction was carried out in an open 10 mL double walled glass vial, which was cooled to 10 °C using a microwave transparent cooling liquid. The temperature was measured with a fiber-optic device inserted into the reaction vessel. IR spectra were recorded in KBr disks using a Perkin-Elmer System 2000 FT-IR spectrophotometer. ^1^H-NMR (400 MHz) and ^13^C-NMR (100 MHz) spectra were recorded on a Bruker DPX 400, super-conducting NMR spectrometer in CDCl_3_ or DMSO-*d_6_* as solvent and TMS as internal standard; chemical shifts were reported in δ units (ppm). Mass spectra were measured on a VG Autospec-Q (high resolution, high performance, tri-sector GC/MS/MS). Microanalyses were performed on a LECO CHNS-932 Elemental Analyzer.

### 2-(4-Dimethylamino-2-oxo-but-3-enyl)isoindole-1,3-dione (**2**)

Thermal method: A mixture of phthalimidoacetone (2.03 g, 0.01 mol) and DMFDMA (1.19 g, 0.01 mol) in xylene (5 mL) was heated under reflux for 8 h. The solid product was collected by filtration and crystallized from EtOH. The reaction gave yellow crystal, yield (76%); mp 162 °C (lit. [11] mp 159–162 °C ); IR (KBr) ν 1,769, 1,714, 1,660 (CO) (cm^-1^); MS (EI) *m*/z (%): 258 [M^+^, 20%], 160(20), 76(10); ^1^H-NMR (DMSO-*d_6_*): δ(ppm) 2.72 (s, 3H, CH_3_), 3.05 (s, 3H, CH_3_), 4.40 (s, 2H, CH_2_), 5.04 (d,1H, *J* = 12 Hz, CH), 7.61 (d,1H, *J* = 12 Hz, CH), 7.85-7.91 (m, 4H, phthalimidyl-H); Anal. Calcd. for C_14_H_14_N_2_O_3_: C, 65.11; H, 5.46; N, 10.85; Found: C, 65.18; H, 5.44; N, 10.76.

Microwave method: A mixture of phthalimidoacetone (2.03 g, 0.01 mol) and DMFDMA (1.19 g, 0.01 mol) was irradiated by focused microwave at 180 °C for 20 min. The build-up of pressure in the closed reaction vessel was carefully monitored. After the irradiation, the reaction tube was cooled with high-pressure air through an inbuilt system in the instrument until the temperature had fallen below 50 °C. The solid product, so formed, was collected by filtration and crystallized from EtOH to give compound **2** in 77% yield.

### General procedure for the synthesis of compounds ***5*** and ***6***

A solution of compound **2** (2.58 g 0.01 mol), benzaldehyde (1.06 g 0.01 mol), urea (0.76 g 0.01 mol), and acetic acid (10 mL) was heated under reflux for 3 h (completion of reaction was monitored by TLC). The precipitate thus formed was then collected by filtration, washed with water and crystallized from acetic acid to give (61%) of tetrahydropyrimidine **5**. The filtrate was then washed thoroughly with water the solid product, so formed, was collected by filtration and crystallized from ethanol to afford dihydropyridine **6**.

*2-[2-Oxo-2-(2-oxo-4-phenyl-1,2,3,4-tetrahydropyrimidin-5-yl)ethyl]isoindole-1,3-dione* (**5**): Yellow crystals, yield 61%; mp 280–282 °C; IR (KBr, cm^-1^): 3,236, 3,101 (NH), 1,714, 1,655 (CO); MS (EI) m/z: (%) 361 [M+, 15%]; 342 (100), 316 (22), 200 (86), 172 (25). 157 (100), 131 (76), 103 (38), 77 (52); ^1^H-NMR (DMSO-*d_6_*): The diasterotopic protons NCH_2_ appear as two doublets 4.81, 4.91 (d, 1H, *J* = 13.6 Hz, d, 1H, *J* = 13.6 Hz, NCH_2_), 5.15 (s, 1H, CH), 7.23 (t, 3H, *J* = 8.0 Hz, arom-H), 7.31 (t, 2H, *J* = 7.2 Hz, arom-H), 7.83-7.90 (m, 6H, arom-H, NH), 9.74 (s, 1H, NH); ^13^C-NMR (DMSO-*d_6_*): δ = 187.41, 168.08, 152.09 (CO), 144.41, 140.92, 135.19, 132.80, 128.88, 127.84, 126.72, 123.70, 110.84, 53.20, 42.85 (CH_2_); Anal. Calcd. for C_20_H_15_N_3_O_4_ (361.35): C, 66.48; H, 4.18; N, 11.63. Found: C, 66.49; H, 4.40; N, 11.54.

2-[2-(5-(2-(1,3-Dioxoisoindolin-2-yl)acetyl)-4-phenyl-1,4-dihydropyridin-3-yl)-2-*oxoethyl]isoindoline-1,3-dione* (**6**): Orange crystals, yield 20%; mp 274–276 °C; IR (KBr, cm^-1^): 3,236, 3,101 (NH), 1,714, 1,655 (CO); MS (EI) m/z: (%) 531 [M+, 14%]; 513 (100), 454 (32), 369 (55), 343 (22). 284 (18), 182 (26), 160 (100), 147 (72), 104 (74), 76 (61); ^1^H-NMR (DMSO-*d_6_*): 4.76-4.90 (m, 4H, 2NCH_2_), 5.16 (s, 1H, CH), 7.10 (t, 3H, *J* = 7.6 Hz, arom-H), 7.16 (d, 2H, *J* = 7.2 Hz, arom-H), 7.21-7.37 (m, 2H, arom-H), 7.87-7.97 (m, 8H, arom-H), 10.14 (s, 1H, NH); ^13^C-NMR (DMSO-*d_6_*): δ = 188.41, 167.62 (CO), 146.11, 138.00, 134.68, 131.58, 128.80, 128.44, 126.28, 126.09, 123.26, 114.01, 27.24, 42.80 (CH_2_); Anal. Calcd. for C_31_H_21_N_3_O_6_ (531.51): C, 70.05; H, 3.98; N, 7.91. Found: C, 70.21; H, 4.20; N, 8.13.

### 2-(2-3,5-Di[2-(1,3-dioxo-2,3-dihydro-1H-2-isoindolyl)acetyl]phenyl-2-oxoethyl)-1,3-isoindolinedione (**9**)

Thermal method: A mixture of compound **2** (2.58 g, 0.01 mol) and acetic acid (10 mL) was refluxed for 10 h, upon completion of the reaction, as checked by TLC, the product was extracted with EtOH. After evaporation of the solvent under reduced pressure, the product was recrystallized from toluene. This compound was obtained as light brown crystals, yield (84 %); mp. 300–301 °C; IR (KBr, cm^-1^): 1,776, 1,721, (CO); MS (EI) m/z (%) = 639 [M^+^]; ^1^H-NMR (DMSO-*d_6_*): δ (ppm) 5.57 (s, 6H, CH_2_), 7.93-7.99 (m-12H, phthalimidyl-H), 8.98 (s, 3H, benzyl-H); ^13^C-NMR (DMSO-*d_6_*): δ 192.23, 167.98 (2 CO), 135.33, 135.20, 133.39, 132.02, 123.93, 45.50 (CH_2_); Anal. Calcd. for C_36_H_21_N_3_O_9_ (639.56); C, 67.61; H, 3.31; N, 6.57. Found 67.46; H, 3.33; N, 6.81.

Microwave method: A mixture of compound **2** (2.58 g, 0.01 mol), zeolite (Montmorillonite k-10 clay, 1 g) and acetic acid (1 mL) was irradiated in microwave at 150 ºC for 60 min. The crude product was poured onto water, the solid product, so formed, was collected by filtration and crystallized from toluene. This gave compound **9** in 57% yield.

### General procedure for the synthesis of compounds ***11a*-*c***

A solution of compound **2** (2.58 g 0.01 mol), compounds **10a-c** (0.01 mol), and EtOH (20 mL) was heated under reflux for 5 h (completion of reaction was monitored by TLC). The reaction mixture was poured into cold water, filtered and crystallized from ethanol to afford compounds **11a-c**.

*2-(2-Oxo-4-phenylamino-but-3-enyl)isoindole-1,3-dione* (**11a**): Yellow-orange crystals, yield 88%; mp 167–169 °C; IR (KBr, cm^-1^): 3,457 (NH), 1,773, 1,706, 1,640 (CO); MS (EI) m/z: (%) 306 [M+, 20%]; 160 (25), 146 (100), 117 (8), 104 (14), 77 (12); ^1^H-NMR (DMSO-*d_6_*): 4.50 (s, 2H, NCH_2_), 5.45 (d, 1H, *J* = *8.0* Hz, CH), 7.03 (t, 1H, *J = 7.2* Hz, phenyl-H), 7.25 (d, 2H, *J =* 8.0 Hz, phenyl-H), 7.30 (t, 2H, *J* = 7.2 Hz, phenyl-H), 7.77 (dd, 1H, *J* = 8.0 Hz, *J* = 8.0 Hz CH), 7.87-7.90 (m, 2H, phthalimidyl-H), 7.91-7.95 (m, 2H, phthalimidyl-H), 11.15 (d, 1H, *J* = 12.6 Hz, NH); ^13^C-NMR (DMSO-*d_6_*): δ = 191.47, 168.22 (CO), 145.75, 140.38, 135.16, 132.14, 130.06, 123.96, 123.69, 116.66, 93.58, 45.79 (NCH_2_); Anal. Calcd. for C_18_H_14_N_2_O_3_ (306.31): C, 70.58; H, 4.61, N, 9.15. Found: 70.55; H, 4.61, N, 9.27.

*2-[4-(4-Methoxyphenylamino)-2-oxo-but-3-enyl]isoindole-1,3-dione* (**11b**): Yellow crystals, yield 83%; mp 204–206 °C; IR (KBr, cm^-1^): 3,455 (NH), 1,772, 1,706, 1,632 (CO); MS (EI) m/z: (%) 336 [M+, 82%]; 175 (100), 160 (42), 132 (65), 103 (22), 76 (21); ^1^H-NMR (DMSO-*d_6_*): 3.71 (s, 3H, OCH_3_), 4.46 (s, 2H, NCH_2_), 5.37 (d, 1H, *J = 7.8* Hz, CH), 6.88 (d, 2H, *J = 8.4* Hz, arom-H), 7.19 (d, 2H, *J = 8.4* Hz, arom-H), 7.64 (dd, 1H, *J = 7.8* Hz, *J* = 7.8 Hz CH), 7.86-7.89 (m, 2H, phthalimidyl-H), 7.91-7.93 (m, 2H, phthalimidyl-H), 11.18 (d, 1H, *J* = 12.6 Hz, NH); ^13^C-NMR (DMSO-*d_6_*): δ = 190.27, 167.70 (CO), 155.80, 145.99, 134.62, 133.30, 131.65, 123.18, 117.65, 114.77, 92.08, 55.99 (NCH_2_), 44.34 (OCH_3_); Anal. Calcd. for C_19_H_16_N_2_O_4_ (336.34): C, 67.85; H, 4.79, N, 8.33. Found: C, 67.73; H, 4.85, N, 8.30.

*2-[4-(4-Nitrophenylamino)-2-oxo-but-3-enyl]isoindole-1,3-dione* (**11c**): Yellow crystals, yield 79%; mp 234–236 °C; IR (KBr, cm^-1^): 3,460 (NH), 1,768, 1,715, 1,653 (CO); MS (EI) m/z: (%) 351 [M+, 84%]; 306 (53), 261 (28), 191 (60), 159 (41), 145 (43), 116 (45), 104 (70). 76 (100); ^1^H-NMR (DMSO-*d_6_*): 4.57 (s, 2H, NCH_2_), 5.65 (d, 1H, *J =* 8.4 Hz, CH), 7.49 (d, 2H, *J =* 9.0 Hz, arom-H), 7.82 (dd, 1H, *J =* 8.4 Hz, *J* = 8.4 Hz CH), 7.85-7.89 (m, 2H, phthalimidyl-H), 7.90-7.94 (m, 2H, phthalimidyl-H), 8.15 (d, 2H, *J =* 9.0 Hz, arom-H), 11.18 (d, 1H, *J* = 12.6 Hz, NH); ^13^C-NMR (DMSO-*d_6_*): δ = 190.26, 167.32 (CO), 155.72, 145.42, 141.53, 134.57, 131.69, 125.63, 123.32, 116.14, 108.33, 36.55 (NCH_2_); Anal. Calcd. for C_18_H_13_N_3_O_5_ (351.31): C, 61.54; H, 3.73, N, 11.96. Found: C, 61.53; H, 3.82, N, 11.96.

### General procedure for the synthesis of ***12*** and ***13***

Equimolar quantities (0.01 mol) of either compound **2** or **11a** and corresponding zeolite were heated under solvent-free conditions for 30 min in a focused microwave oven at 160 °C. Upon completion of the reaction, as checked by TLC, the product was extracted with EtOH (3 × 10 mL). After evaporation of the solvent under reduced pressure, the product was recrystallized from toluene.

*2-Phenylisoindole-1,3-dione* (**12**): Buff crystals, yield 96%; mp 206-208 °C (lit. [13] mp 205–207 °C yield 95%); MS (EI) m/z: (%) 223 [M+, 100%]; 179 (78), 152 (10), 104 (16), 76 (40). ^1^H-NMR (DMSO-*d_6_*): 7.44 (d, 3H, *J =* 7.2 Hz, phenyl-H), 7.53 (t, 2H, *J =* 7.2 Hz, phenyl-H), 7.91-7.94 (m, 2H, phthalimidyl-H), 7.96-7.99 (m, 2H, phthalimidyl-H); ^13^C-NMR (DMSO-*d_6_*): δ = 167.49 (CO), 135.17, 132.37, 132.02, 129.31, 128.53, 127.89, 123.89; Anal. Calcd. for C_14_H_9_NO_2_ (223.23): C, 75.33; H, 4.06, N, 6.27. Found: C, 75.57; H, 4.12, N, 6.36.

*2-Methyl-isoindole-1,3-dione* (**13**): Buff crystals, yield 87%, mp 131–133 °C (lit. [14] mp 134 °C and yield 68%); MS (EI) m/z: (%) 161 [M+, 100%]; 132 (50), 117 (74), 104 (82), 76 (81), 66 (44); ^1^H-NMR (DMSO-*d_6_*): 3.36 (s, 3H, CH_3_), 7.81-7.83 (m, 2H, phthalimidyl-H), 7.84-7.88 (m, 2H, phthalimidyl-H); ^13^C-NMR (DMSO-*d_6_*): δ = 168.53 (CO), 134.72, 132.30, 123.36, 24.20 (CH_3_); Anal. Calcd. for C_9_H_7_NO_2_ (161.16): C, 67.07; H, 4.38, N, 8.69. Found: C, 67.01; H, 4.39, N, 9.19.

### Reaction of phthalic anhydride with aniline

Equimolar quantities (1.48 g, 0.01 mol) of phathalic anhydride and aniline (0.93 g, 0.01 mol) were heated for 60 min in a focused microwave oven at 180 °C under solvent-free conditions. Upon completion of the reaction, as checked by TLC, the product was extracted with EtOH. After evaporation of the solvent under reduced pressure, the product was recrystallized from toluene to afford compound **12**, yield 94%.

### 2-[2-(5-Hydroxynaphtho[1,2-b]furan-3-yl)-2-oxoethyl]isoindole-1,3-dione (***18***)

Thermal method: A mixture of compound **11a** (3.06 g, 0.01 mol) and naphthoquinone (1.54 g, 0.01 mol) was dissolved in glacial acetic acid (10 mL), then stirred overnight at room temperature. The so formed crystals were collected by filtration and crystallized from dioxane. This compound was obtained in 68 % yield, mp 303 °C (lit. [15] mp 301–303 °C); IR (KBr, cm^-1^): 3,309 (OH), 1,776, 1,722, 1,666 (CO); MS (EI) m/z (%) = 371 [M^+^]; ^1^H-NMR (DMSO-*d_6_*): δ 5.17 (s, 2H, CH_2_), 7.45 (s, 1H, H-4), 7.58 (dt, 1H, *J* = 7.8, 1.2 Hz, H-7), 7.70 (dt, 1H, *J* = 7.8, 1.2 Hz, H-8), 7.91-7.92 (m, 1H, H-14), 7.97-7.98 (m, 1H, H-13), 8.23 (d, 1H, *J =* 8.4 Hz, H-9), 8.25 (d, 1H, *J* = 8.4 Hz, H-6), 9.31 (s, 1H, H-2), 10.34 (s, 1H, OH); ^13^C-NMR (DMSO-*d_6_*): δ 188.71 (C-10), 167.99 (C-12), 153.74 (C-2), 151.65 (C-5), 145.02 (C-9b), 135.28 (C-14), 132.02 (C-12a), 128.01 (C-8), 125.61 (C-7), 123.97 (C-5a), 123.89 (C-13), 123.80 (C-6), 121.19 (C-9a), 120.28 (C-3a), 120.08 (C-3), 119.85 (C-9), 100.12 (C-4), 45.23 (CH_2_, C-11); Anal. Calcd. for C_22_H_13_NO_5_ (371.35); C, 71.16; H, 3.53; N, 3.77. Found C, 71.14; H, 3.65; N, 3.95.

Microwave method: A mixture of compound **11a** (3.06 g, 0.01 mol), naphthoquinone (1.54 g, 0.01 mol), and glacial acetic acid (1 mL) was irradiated at 30 °C for 30 min, continuously at the maximum power of 80 W. After completion of the reaction the solvent was evaporated. The crude product was then collected by filtration and crystallized from dioxane to give the title compound in 76% yield.

## 4. Conclusions

In summary, we could show that the enaminone **2** is a valuable precursor to the tetrahydropyrimidine, dihydropyridine, triacylbenzene and naphthofuran moieties. It is worthwhile to mention here that thermal and microwave assisted reactions were conducted in different solvents with exception of **2**, **12** and **13**, which were prepared by microwave heating without solvent. We have also revealed that the reactions described proceeded to completion in a much shorter time when irradiated in a focused microwave oven. Moreover, microwave irradiation in combination with simultaneous cooling assisted the reactions and produced somewhat higher yield than those obtained by conventional heating.
